# Antioxidant and Hepatoprotective Potentiality of *Randia dumetorum* Lam. Leaf and Bark via Inhibition of Oxidative Stress and Inflammatory Cytokines

**DOI:** 10.3389/fphar.2016.00205

**Published:** 2016-07-14

**Authors:** Raghuram Kandimalla, Sanjeeb Kalita, Bikas Saikia, Bhaswati Choudhury, Yogendra P. Singh, Kasturi Kalita, Suvakanta Dash, Jibon Kotoky

**Affiliations:** ^1^Drug Discovery Lab, Division of Life Sciences, Institute of Advanced Study in Science and TechnologyGuwahati, India; ^2^Department of Pathology, Hayat HospitalGuwahati, India; ^3^Girijananda Chowdhury Institute of Pharmaceutical ScienceGuwahati, India

**Keywords:** *Randia dumetorum*, antioxidant, liver protection, inflammation, cytokines

## Abstract

*Randia dumetorum* Lam. (RD) (Rubiaceae) is traditionally used by some tribes of Assam and Manipur of North East India for the treatment of liver ailments. In this context, to scientifically validate this indigenous traditional knowledge, we have evaluated the antioxidant and hepatoprotective activity of RD leaf and bark. The methanol extracts of RD leaf and bark were evaluated for *in vitro* antioxidant activity which exhibited good antioxidant activity in terms of reducing power assay, total antioxidant assay and DPPH (1,1-diphenyl-2-picrylhydrazyl) radical scavenging assay. Total phenolic and flavonoid content were found to be 112 ± 3.24 mg and 138 ± 2.46 mg gallic acid equivalents/g extract and 2.6 ± 0.26 mg and 3.34 ± 0.31 mg rutin equivalents/g extract respectively for RD leaf and bark methanol extracts. The *in vivo* hepato protective activity of the RD leaf and bark extract was evaluated against carbon tetrachloride (CCl_4_) induced hepatic damage in male wistar rats. CCl_4_ administration induced hepatic damage in rats resulted in increased levels of aspartate transaminase, alanine transaminase, alkaline phosphatase, lactate dehydrogenase, thiobarbituric acid reacting substances, albumin, bilirubin, TNF-α, IL-1β and decreased levels of total protein and antioxidant enzymes like superoxide dismutase, catalase, and glutathione reductase. RD leaf and bark methanol extracts pre-treatment exhibited protection against CCl_4_ induced hepatotoxicity by reversing all the abnormal parameters to significant levels. Histopathological results revealed that RD leaf and bark extracts at 400 mg/kg protects the liver from damage induced by CCl_4_. The results of this study scientifically validate the traditional use of RD leaf and bark for the treatment of liver ailments.

## Introduction

Oxidation is the key process for production of energy in an organism to fuel biological processes (Kong et al., 2010). Over production of oxygen free radicals increase the oxidative stress in organisms by causing deleterious effects to cell structures. Many disease conditions like cancer, diabetes, atherosclerosis, hypertension, neurodegenerative diseases, aging, inflammation, and acute and chronic liver diseases precipitate directly or indirectly through reactive oxygen species (ROS) mediated mechanisms (Liang et al., 2011; Sarwar et al., 2015). Antioxidants are the compounds that neutralize the free radicles produced in the body (Fu et al., 2010). Liver is second largest organ in human body which plays a major role in metabolism. Impairment of liver leads to serious health consequences and in the majority of cases it is life threatening. Management of liver diseases is still a challenge to the modern medicine with a few treatment options are available. Oxidative stress on liver affects the antioxidant enzymes like superoxide dismutase (SOD), catalase (CAT), and glutathione reductase (GSH) and increase the lipid peroxidation (LPO) in liver (Banu et al., 2012; Habib et al., 2015). Different poly-herbal formulations are often prescribed by traditional practitioners as hepatoprotective agents. These formulations contain combination of natural antioxidants which act synergistically together to protect the liver cells.

*Randia dumetorum* Lam. (RD) is a large thorny shrub belongs to the family Rubiaceae. This plant is widely available throughout India and African subcontinent up to 4000 feet altitude. RD was reported to contain mannitol, iridoid-10-methylixoside, coumarin glycosides, triterpenoid glycosides, randianin and saponins named as dumentoronin A, B, C, D, E, F, etc. It also used in ayurvedic medicines for having properties like rasa, guna, virya, vipaka, etc. RD was also reported for its immunomodulatory, analgesic, antiinflammatory, antiallergic, and antibacterial activities (Patel et al., 2011). In North Eastern region of India some tribes of Assam and Manipur uses RD fruit, bark and leaf for treatment of different liver ailments. It is the need of the hour to scientifically validate this traditional knowledge before it gets lost. In this present study we evaluated the antioxidant and hepatoprotective activities of RD leaf and bark methanol extracts on CCl_4_ induced acute liver damage in rat. Successful validation of the claimed properties of RD can potentially lead to development of therapeutics for liver ailments.

## Materials and Methods

### Drugs and Chemicals

1,1-Diphenyl-2-picrylhydrazyl was obtained from Sigma (Sigma-Aldrich, USA). CCl_4_, BHT and Ascorbic acid were obtained from Merck, India. Silymarin was obtained from Micro Labs Limited. Biological kits were obtained from Accurex, India. The entire chemicals used in the experiments were of analytical grade and Millipore water was used throughout the experiments.

### Preparation of the Plant Extract

Leaf and bark of RD were collected from Kamrup district of Assam, India in the month of January. Plant was identified by a botanist at North East India Ayurvedic Institute (NEIAI), Guwahati and a voucher Specimen (no IASST/1016) was deposited in IASST, herbarium library. Plant material was dried under shade and after drying leaf and bark grinded into a coarse powder. Plant material (100 g) was soaked in methanol (500 ml) for 4 days and filtered using Whatman filter paper. The methanol used for extraction was completely evaporated by rotary evaporator at 45°C to avoid toxicity. The percentage yield was calculated 10–15% for RD leaf and 5–7% for RD bark.

### Determination of Total Phenolic and Flavonoid Content

The total phenolic content of the extracts were determined using the Folin-Ciocalteu assay (Sabir and Rocha, 2008). The results are expressed as grams of gallic acid equivalents per 100 g of dry extract. The total flavonoid content was determined with a colorimetric assay (Moreno et al., 2000) using rutin as a standard. The results are expressed as grams of rutin equivalents per 100 g of dry extract.

### *In Vitro* Antioxidant Activity

#### DPPH Radicle Scavenging Assay

The radicle scavenging activity was evaluated by modified DPPH assay (He et al., 2012). Briefly to 2.7 mL of 0.2 mM DPPH, 0.3 mL of the extract solution at various concentrations was added. The mixture was shaken vigorously and incubated at room temperature for 1 h before the absorbance was measured at 517 nm. The radical scavenging activity was calculated as follows: scavenging rate = [(As - Ai)/As] × 100, where As is the absorbance of pure DPPH and Ai is the absorbance of DPPH in the presence of various extracts. Ascorbic acid at different concentrations identical to the experimental samples was used as reference.

#### Reducing Power Assay

The reducing power of the extracts was estimated using the standard method (Oyaizu, 1986). Briefly, increasing concentrations of 0.2 mL of extracts were mixed with 2.5 mL of phosphate buffer (0.2 M, pH 6.6) and 2.5 mL of potassium ferricyanide (1%). After incubation at 50°C for 20 min, 2.5 mL of trichloroacetic acid (10%) was added and each mixture was centrifuged at 1000 rpm for 10 min. Then, 2.5 mL of the supernatant was collected and mixed with 2.5 mL of deionized water and 0.5 mL of ferric chloride (0.1%). The absorbance was measured at 700 nm. The increased absorbance of the reaction mixture indicated increased reducing power. BHT was used as a standard for comparison.

### Total Antioxidant Activity of Plant Extracts

Measurement of total antioxidant capacity of plant extracts were performed by photochemiluminescence method in the Photochem instrument, Germany. Lipid soluble antioxidant capacity assay estimate the photochemical generation of free radicals with a sensitive detector by using chemiluminescence. Free radicals were produced from the luminal, which worked partly as a photosensitizer and oxygen radical detection reagent. The lipophilic antioxidants were measured with the kits commercially available from Photochem (Germany). Total antioxidant activity of plant extracts was expressed in trolox equivalents (Kalita et al., 2016).

### Acute Toxicity Studies

Acute oral toxicity studies were performed according to OECD guidelines to test chemicals. Adult swiss albino mice of either sex (six animals) were used for this study. Animals were kept fasting overnight with free access to water. Next day morning single dose of RD methanol leaf and bark extracts at 2000 mg/kg body weight were administered orally to three animals each. After administrating the single large dose of plant extract, animals were observed for different adverse effects for 14 days. If mortality was observed in two out of three animals, then the dose was identified as toxic dose. If mortality was observed in 1 animal, experiment was repeated again with the same dose to confirm the toxic dose. If mortality observed again experiment was continued with low doses (300, 50, and 5 mg/kg body weight).

### Hepato Protective Effect of Methanol Leaf and Bark Extracts of RD

#### Animals

Adult male wistar rats (150–200 g) were obtained from the institutional animal house of Institute of Advanced Study in Science and Technology (IASST), Guwahati (India). Animals were maintained at 24°C ± 1°C, with relative humidity of 45–55% and 12:12 h dark/light cycle. The animals had free access to standard pellet diet (Provimi Animal Nutrition, Pvt, Ltd, India) and water throughout the experimental protocol. All experiments were carried out between 09:00 and 17:00 h. The experimental protocol was approved by the Institutional Animal Ethics Committee (IAEC) of IASST, Guwahati (CPCSEA/44/2014) and performed in accordance with the guidelines of Committee for Control and Supervision of Experimentation on Animals (CPCSEA), Government of India on animal experimentation.

#### Experimental Design

Total of 42 animals were divided into seven groups of six animals each.

• Group I served as normal control and received D.W for 14 days orally and on 14th day olive oil (1.5 ml/kg, i.p.).
• Group II served as toxic control and received 0.3% CMC for 14 days orally and on 14th day CCl_4_ (1.5 ml/kg, i.p.) in 1:1 dilution with olive oil.
• Group III served as standard group and received silymarin (100 mg/kg) for 14 days orally and on 14th day CCl_4_ (1.5 ml/kg, i.p.) in 1:1 dilution with olive oil.
• Groups IV and V served as treatment groups and received RD methanol leaf extract (200 and 400 mg/kg) for 14 days orally and on 14th day CCl_4_ (1.5 ml/kg, i.p.) in 1:1 dilution with olive oil.
• Groups VI and VII served as treatment groups and received RD methanol bark extract (200 and 400 mg/kg) for 14 days orally and on 14th day CCl_4_ (1.5 ml/kg, i.p.) in 1:1 dilution with olive oil.

After 48 h of CCl_4_ administration all the animals were sacrificed and blood and liver was collected for biochemical estimation and histopathology analysis.

#### Serum Biochemical Estimation

Blood was collected from retro-orbital route under mild anesthesia and allowed to clot. Serum was separated by centrifugation at 3000 rpm for 10 min for estimation of aspartate transaminase (AST), alanine transaminase (ALT), alkaline phosphatase (ALP), lactate dehydrogenase (LDH), albumin, total protein, urea, total and direct bilirubin by commercially available kits as per the instructions given by the manufacturer (Accurex, India). ELISA kits from R&D systems, USA were used to measure the serum pro inflammatory cytokines TNF-α and IL-1β. Each sample was done in duplicate and results were expressed in Pg/ml (Kandimalla et al., 2016b,c).

#### Tissue Biochemical Assays

All the treatments were done at 4°C. Liver homogenate was prepared with 50 mM cold potassium phosphate buffer (pH 7.4). The resulting suspension was centrifuged at 3000 rpm for 15 min and supernatant was collected and analyzed for SOD (Marklund and Marklund, 1974), CAT (Aebi, 1974), GSH (Ellman, 1959), and thiobarturic acid reacting substances (TBARSs; Xia et al., 2013).

#### Histopathology Examination

Livers was collected in 10% buffered formaldehyde and preserved for at least 24 h. Further liver samples were dehydrated gradually with ethanol (70–100%), cleared in xylene and embedded in paraffin. Sections of 5 μm were prepared by microtome assembly (Leica, Germany) and stained with hematoxylin and eosin to examine under microscope (10x) for histopathological changes (Kalita et al., 2015; Bhardwaj et al., 2016; Choudhury A.J. et al., 2016).

### Statistical Analysis

All the results were expressed as mean ± SD. One way ANOVA followed by Tukey’s multiple comparison test was used to compare the different parameters between the groups. A *p*-value < 0.05 was considered as significant.

## Results

### Total Phenolic and Flavonoid Content

Presence of phenolic type compounds can be correlates with the antioxidant activity of medicinal plants (Bravo, 1998; Huang et al., 2010; Chen et al., 2011). In this study, the total phenolic and flavonoid content of the methanol extract of RD leaf and bark was determined (**Table 1**). Total phenolic and flavonoid content were found to be 112 ± 3.24 mg and 138 ± 2.46 mg gallic acid equivalents/g extract and 2.6 ± 0.26 mg and 3.34 ± 0.31 mg rutin equivalents/g extract respectively for RD leaf and bark methanol extracts. It is observed that RD bark methanol extract contain more total phenolic and flavonoid content than RD leaf methanol extract.

**Table 1:** Total phenolic, flavonoid, and antioxidant content of methanol extracts of RD leaf and bark.

Sl.no	Plant extract	Total phenolic content by gallic acid equivalents (mg)	Total flavonoid content by rutin equivalents (mg)	Total antioxidant activity by Trolax equivalents (nmol)
1	RD (Leaf)	112 ± 3.24	2.6 ± 0.26	4.912
2	RD (Bark)	138 ± 2.46	3.34 ± 0.31	6.049

*All the results were expressed in mean ± SD (n = 3). RD, Randia dumetorum; Sl.no, serial number, which represents the number of extracts*.

### *In Vitro* Antioxidant Activity

#### DPPH Radical Scavenging Activity

**FIGURE 1:**
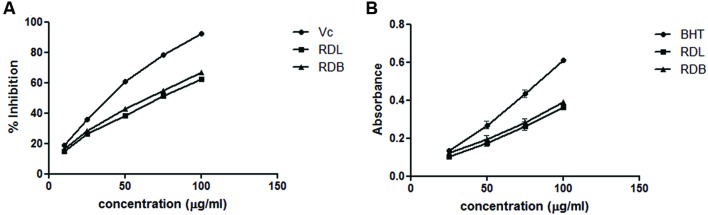
**(A)** 1,1-Diphenyl-2-picrylhydrazyl (DPPH) radicle scavenging assay of methanol extract of RD leaf and bark extracts: (RDL), RD leaf extract; (RDB), RD bark extract; (Vc), ascorbic acid. **(B)** Reducing power activity of methanol extract of RD leaf and bark extracts: RDL, RD leaf extract; RDB, RD bark extract; BHT, butylated hydroxytoluene.

1,1-Diphenyl-2-picrylhydrazyl is a stable, organic free radical widely used to detect the scavenging activity of the antioxidants (Debnath et al., 2015). The DPPH scavenging activity of methanol extracts of RD leaf and bark at different concentrations are presented in **Figure 1A**. Both the extracts show dose dependent DPPH scavenging activity. Apparently ascorbic acid showed the highest radical scavenging activity than methanol plant extracts.

#### Reducing Power Ability

Reducing power ability of the compounds is responsible for the potent antioxidant activity of the extracts (Choudhury B. et al., 2016). The ability of RD extracts to convert Fe^3+^ to Fe^2+^ was considered as reducing potential. Formation of Fe^2+^ can be monitored by measuring the prussian blue color formation at 700 nm. The reducing ability of methanol extracts of RD leaf and bark were increased with increasing the concentrations (**Figure 1B**). The order of reducing ability of the tested sample is BHT > RD bark > RD leaf.

#### Total Antioxidant Activity

Photo-chemi-luminescence method in the Photochem instrument was dependent on the antioxidant concentration based on the Guldberg-Waage law. This law expresses the magnitude of the reaction. Reactions of radicals and antioxidants give relatively stable products which are stable and measurable. Antioxidant capacity of the drug/substance depends on the concentration (Hic and Balic, 2012). Total antioxidant activity of methanol leaf and bark extracts of RD was presented in **Table 1**.

#### Acute Toxicity Studies

No observed adverse effect level (NOAEL) dose is the highest exposure drug dose at which there is no significant increase in adverse effects compared to non-treated animals. In this study RD leaf and bark methanol extracts at 2000 mg/kg did not show any effect on heart rate, respiratory rate, salivation, body temperature, locomotor activity, corneal reflex, body tone, skin tone, abdominal tone, grip strength. tremors, tail elevation, piloerection, twitches, and convulsions are also not observed. After 14 days of observation period no mortality was observed in tested mice. RD leaf and bark methanol extracts at 2000 mg/kg is considered as NOAEL. According to OECD guidelines 2000 mg/kg is the highest dose for acute toxicity studies. RD methanol leaf and bark extracts were safe at highest dose, so no further low dose was tested. 1/10th (200 mg/kg) and 1/5th (400 mg/kg) of NOAEL dose was selected for the present study.

#### Effect of RD Leaf and Bark Methanol Extracts on Serum Biochemical Parameters

Carbon tetrachloride treatment in rats (Group II) showed significant increase in AST, ALT, ALP, LDH, albumin, total bilirubin, direct bilirubin and significant decrease in total protein levels compared to control animals (Group I). Pre-treatment with RD leaf and bark methanol extracts at 200 and 400 mg/kg for 14 days (Groups III–VII) before CCl_4_ intoxication showed significant protection against CCl_4_ by means of decreased serum AST, ALT, ALP, LDH, albumin, total and direct bilirubin and increased total protein levels compared to the CCl_4_ alone treated group (Group II). Though, this result indicated the hepatoprotective ability of test extracts and standard drug silymarin; but the biochemical parameters did not reach to that of control animals (Group I). RD leaf and bark at 200 mg/kg showed less protection than 400 mg/kg treated group. Order of showing hepatoprotective response by means of serum biochemical levels were Group 3 > Group 7 > Group 5 > Group 6 > Group 4 (**Tables 2A**,**B**).

**Table 2A:** Effect of methanol extracts of RD leaf and bark on rat serum enzyme levels.

Sl.no	Group	AST (IU/l)	ALT (IU/l)	ALP (IU/l)	LDH (U/l)
1	Control	44.27 ± 2.97	38.48 ± 2.64	94.83 ± 4.46	424.53 ± 11.65
2	CCl_4_ treatment	127.67 ± 7.59^#^	116.49 ± 8.24^#^	197.58 ± 9.14^#^	992.74 ± 19.38^#^
3	Silymarin (100 mg/kg)	58.66 ± 5.24^∗#^	48.26 ± 3.75^∗#^	108.29 ± 6.81^∗#^	461.44 ± 13.72^∗#^
4	RD leaf 200 mg/kg	113.28 ± 5.32^#^	103.84 ± 4.62^∗#^	181.71 ± 6.84^∗#^	901.26 ± 18.33^∗#^
5	RD leaf 400 mg/kg	82.44 ± 4.81^∗#^	79.64 ± 5.17^∗#^	135.96 ± 5.72^∗#^	741.36 ± 15.61^∗#^
6	RD bark 200 mg/kg	107.82 ± 5.28^∗#^	97.62 ± 4.84^∗#^	174.37 ± 6.28^∗#^	892.53 ± 17.82^∗#^
7	RD bark 400 mg/kg	74.61 ± 3.62^∗#^	68.52 ± 3.82^∗#^	124.48 ± 5.62^∗#^	672.58 ± 14.95^∗#^

**Table 2B:** Effect of methanol extracts of RD leaf and bark on serum biochemical parameters.

**Sl.no**	**Group**	**Albumin (gm %)**	**Bilirubin total (mg %)**	**Bilirubin direct (mg %)**	**Total protein (mg/dl)**
1	Control	1.64 ± 0.07	0.54 ± 0.06	0.14 ± 0.02	7.46 ± 0.59
2	CCl_4_ treatment	3.56 ± 0.66^#^	2.38 ± 0.32^#^	0.97 ± 0.09^#^	3.47 ± 0.58^#^
3	Silymarin (100 mg/kg)	1.76 ± 0.11^∗#^	0.76 ± 0.07^∗#^	0.18 ± 0.02^∗#^	6.36 ± 0.84^∗#^
4	RD leaf 200 mg/kg	2.86 ± 0.54^∗#^	1.84 ± 0.09^∗#^	0.78 ± 0.08^∗#^	4.51 ± 0.67^∗#^
5	RD leaf 400 mg/kg	2.51 ± 0.52^∗#^	1.19 ± 0.08^∗#^	0.47 ± 0.07^∗#^	5.26 ± 0.82^∗#^
6	RD bark 200 mg/kg	2.75 ± 0.57^∗#^	1.71 ± 0.07^∗#^	0.69 ± 0.06^∗#^	4.84 ± 0.58^∗#^
7	RD bark 400 mg/kg	2.23 ± 0.43^∗#^	0.98 ± 0.05^∗#^	0.36 ± 0.07^∗#^	5.58 ± 0.74^∗#^

#### Effect of RD Leaf and Bark Methanol Extract on Serum Cytokine Levels

**FIGURE 2:**
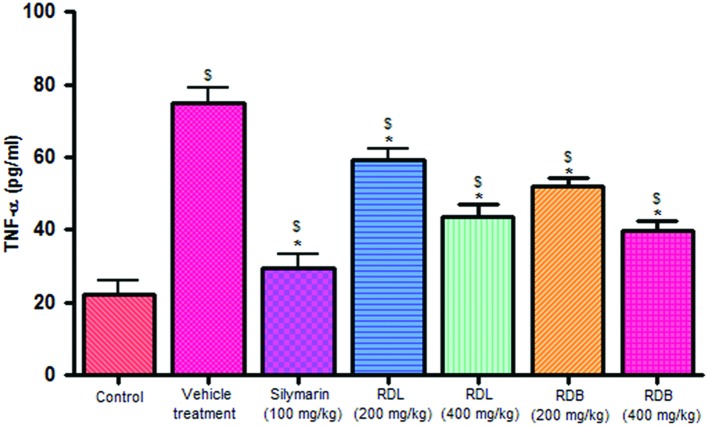
**Effect of different drug treatment on serum TNF-α levels.** All the results were expressed in mean ± SD (*n* = 6). ^$^*p* < 0.05 in comparison with normal animals. ^∗^*p* < 0.05 in comparison with CCl_4_ alone treated animals. RDL, *R. dumetorum* leaf methanol extract; RDB, *R. dumetorum* bark methanol extract.

**FIGURE 3:**
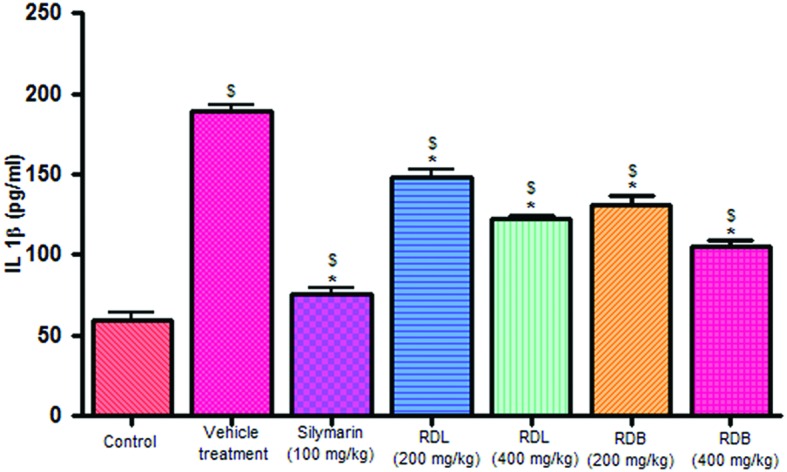
**Effect of different drug treatment on serum IL-1β levels.** All the results were expressed in mean ± SD (*n* = 6). ^$^*p* < 0.05 in comparison with normal animals. ^∗^*p* < 0.05 in comparison with CCl_4_ alone treated animals. RDL, *R. dumetorum* leaf methanol extract; RDB, *R. dumetorum* bark methanol extract.

Carbon tetrachloride induction activates inflammatory cytokines like TNF-α and IL-1β levels in *in vivo* system. Pre-treatment with standard drug silymarin, RD leaf and RD bark methanol extracts before CCl_4_ induction significantly inhibits the TNF-α (**Figure 2**), IL-1β (**Figure 3**) production dose dependently, but this reduction in levels did not reach up to that of control animals. RD bark extract showed potent response to RD leaf extract.

#### Effect of RD Leaf and Bark Methanol Extracts on Antioxidant Enzyme Levels

Administration of CCl_4_ significantly decreases the SOD, CAT, and GSH levels in comparison to control rats. Pre-treatment with RD leaf and bark methanol extracts showed significant increase in all the enzyme levels in comparison with CCl_4_ alone treated rats. But this observed rise in enzyme level was not as significant as standard drug silymarin treated rats (**Table 2C**). Standard drug and extracts treatment did not completely restore the abnormal parameters as that of control animals. RD bark methanol extract showed more significant results than that of leaf extract.

**Table 2C:** Effect of methanol extracts of RD leaf and bark on liver biochemical parameters.

**Sl.no**	**Group**	**SOD (% activity)**	**CAT (U/mg protein)**	**GSH (nm/mg protein)**	**TBARS (nmol/g tissue)**
1	Control	78.82 ± 4.16	35.68 ± 2.82	24.47 ± 2.04	179.54 ± 9.44
2	CCl_4_ treatment	28.51 ± 2.14^#^	14.68 ± 1.76^#^	09.27 ± 1.12^#^	328.39 ± 11.42^#^
3	Silymarin (100 mg/kg)	67.86 ± 5.39^∗#^	28.72 ± 2.18^∗#^	19.48 ± 1.94^∗#^	186.62 ± 9.58^∗#^
4	RD leaf 200 mg/kg	36.75 ± 4.62^∗#^	19.47 ± 1.32^∗#^	11.96 ± 1.17^∗#^	266.72 ± 10.64^∗#^
5	RD leaf 400 mg/kg	49.56 ± 3.68^∗#^	22.65 ± 2.64^∗#^	15.76 ± 1.49^∗#^	236.48 ± 8.72^∗#^
6	RD bark 200 mg/kg	40.65 ± 4.36^∗#^	20.88 ± 1.86^∗#^	13.58 ± 1.58^∗#^	251.43 ± 9.62^∗#^
7	RD bark 400 mg/kg	55.77 ± 5.16^∗#^	24.58 ± 1.78^∗#^	16.64 ± 1.94^∗#^	224.38 ± 8.18^∗#^

*All the results were expressed in mean ± SD (n = 6). One-way ANOVA, Tukey post hoc: ^#^p ≤ 0.05 vs. normal control (Group I), ^∗^p ≤ 0.05 vs. toxic control (Group II). RD, Randia dumetorum; Sl.no, serial number, which represents the group number.*

#### Effect of RD Leaf and Bark Methanol Extracts on Lipid Peroxidation

Carbon tetrachloride treated rats showed significantly increased TBARS levels when compared with control group. Pre-treatment with RD leaf and bark methanol extracts and standard drug silymarin before CCl_4_ intoxication showed significant decrease in TBARS levels compared with CCl_4_ alone treated rats (**Table 2C**), but this reduction did not reach to that of control animals.

#### Histopathology

**FIGURE 4:**
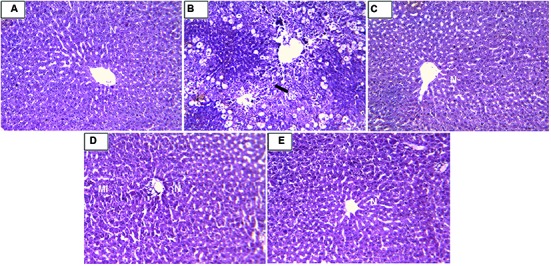
**(A)** Liver of control rat showing normal hepatocytes; x 10. **(B)** Liver of rat treated with CCl_4_ showing formation of septa, bridging necrosis and dense periportal inflammation; x 10. **(C)** Liver of rat treated with CCl_4_ and 100 mg/kg Silymarin showing complete absence of periportal inflammation or necrosis; x 400. **(D)** Liver of rat treated with CCl_4_ and 400 mg/kg of RD methanol leaf extract showing mild periportal inflammation; x 10. **(E)** Liver of rat treated with CCl_4_ and 400 mg/kg RD methanol bark extract showing reversal of changes with absence of periportal inflammation or necrosis; x 10. N, normal hepatocytes; Ne, necrotic cells; MI, mild inflammation.

Histopathology of livers provided supportive evidence for the biochemical analysis. Liver histopathology of rats in control group showed normal hepatocytes (**Figure 4A**). CCl_4_ intoxication causes demolishment of hepatocytes which was evidenced by formation of large septa, collagen accumulation, bridging necrosis, and periportal inflammation (**Figure 4B**). Treatment with standard drug silymarin, RD leaf and bark methanol extracts showed significant protection against CCl_4_ induced hepatic damage. Liver tissue of the treated group showed normal architecture with no necrosis and inflammation (**Figures 4C–E**).

## Discussion

The present study deals with antioxidant and hepatoprotective activities of methanol extract of RD leaf and bark. Phenolic and flavonoid compounds are radical scavengers, metal chelators, reducing agents, hydrogen donors, and singlet oxygen quenchers (Khadri et al., 2010; Saeed and Farzaneh, 2010). Therefore, it is important to measure the total phenolic and flavonoid content of a plant extract to measure the antioxidant capacity. The *in vitro* antioxidant activity of the RD leaf and bark methanol extracts might be due to presence these phenolic and flavonoid component as demonstrated by DPPH scavenging activity, reducing power ability and total antioxidant activity. Liver is the second largest organ of our body involved in vital functions like detoxification of drugs and toxins through metabolism. CCl_4_ is the toxin which produce trichloromethyl radical (CCl_3_•) which is activated by cytochrome P450. This radicle mainly associated with CCl_4_ induced hepatic damage (Shakya et al., 2012; Vuda et al., 2012). These radicals reacts covalently with sulfhydryl-containing proteins in cells which leading to membrane lipid peroxidation and cell necrosis (Muriel et al., 2001).

Serum enzyme levels are significant markers to determine the severity of damage to particular tissue (Tu et al., 2015). CCl_4_ administration cause significant increase in serum enzyme levels like AST, ALT, ALP, LDH, Albumin and decrease levels of total protein levels when compared with control animals which indicate the liver damage. Bilirubin is the product of heme in red blood cells and hyper-bilirubinemia is one of the markers of damaged liver. Treatment with standard drug silymarin and methanol leaf and bark extracts of RD restore the elevated serum levels of AST, ALT, ALP, LDH, Albumin, total and direct bilirubin with increase in total protein levels. Total protein levels also raised in drug treatment groups (Groups III–VII). Restoration of abnormal enzyme levels to normal indicating the protection of RD leaf and bark methanol extracts against the liver injury by CCl_4_.

By-products of LPO cause hepatic damage in hydrophobic core of bio-membranes (Maheswari et al., 2008). Elevated levels of TABRS are observed in CCl_4_ treated rats indicating the formation of free radicals in larger quantity due to failure of antioxidant defense mechanism and hepatic damage. Significant decline in LPO was observed in rats treated with standard drug silymarin and methanol leaf and bark extracts of RD.

We further studied the non-enzymatic antioxidants like GSH and endogenous enzymes like SOD and CAT which involves in neutralizing free radicals. Suppression of these enzyme levels is an indicator for hepatic damage (Ozer et al., 2008). CCl_4_ administration cause decline in all the hepatic antioxidant levels when compared with control animals. Standard drug silymarin and methanol leaf and bark extracts of RD pre-treatment restores the antioxidant levels, which is a positive sign of liver protection. CCl_4_ metabolism stimulated the Kupffer cells which activates this TNF-α and IL-1β. IL-1β is strong inflammatory cytokines which involves in the production of prostaglandins, macrophage activation, and neutrophil infiltration. Activation of inflammatory cytokine cascade produces significant damage to the cells especially to hepatocytes (Edwards et al., 1993; Ishiyama et al., 1995; Kandimalla et al., 2016a). RD leaf and bark treatment to animal before CCl_4_ cause significant protection, which might be due to stabilization of Kupffer cells and inhibition of macrophage activation and prostaglandin production. Histopathology results provided supportive evidence for biochemical analysis. Methanol leaf and bark extract of RD showed significant morphological changes in liver tissue compare to CCl_4_ treated group. Decreased periportal inflammation was also observed in silymarin and extract treated animals. In this study RD bark methanol extract showed highest protection against CCl_4_ induced liver damage than RD leaf methanol extract which is comparable to standard drug silymarin.

## Conclusion

The present study provides a scientific rational for the traditional use of RD leaf and bark for the treatment of liver ailments. RD bark is more effective than the leaf extract in the treatment of liver ailments. Fractionation, isolation and screening of active chemical components from these extracts are in progress in our laboratory. Successful completion of this work can potentially contribute in the development of herbal remedy for liver disorders.

## Author Contributions

Mr. RK is the first author and performed all the *in vitro* and *in vivo* experiments like animal experiments setup, biochemical estimation, and tissue processing etc. Mr. SK, Mr. BS, and Miss. BC helped the first author in different steps of experiments and manuscript preparation. Dr. KK performed the hisopathology of liver and examined for pathological changes. Prof. SD helped in literature survey and study design. Prof. JK contributed towards study design, experimental setup, results supervision, and manuscript correction.

## Conflict of Interest Statement

The authors declare that the research was conducted in the absence of any commercial or financial relationships that could be construed as a potential conflict of interest.
